# Epidemiological, Clinical, and Paraclinic Aspect of Cutaneous Sarcoidosis in Black Africans

**DOI:** 10.1155/2015/802824

**Published:** 2015-11-08

**Authors:** Mamadou Kaloga, Ildevert Patrice Gbéry, Vagamon Bamba, Yao Isidore Kouassi, Elidjé Joseph Ecra, Almamy Diabate, Sarah Kourouma, Kouadio Celestin Ahogo, Kouassi Alexandre Kouamé, Komenan Kassi, Kanga Kouame, Abdoulaye Sangaré

**Affiliations:** ^1^Department of Dermatology and Venereology, Teaching Hospital of Treichville, Abidjan, Côte d'Ivoire; ^2^Department of Dermatology and Venereology, Teaching Hospital of Bouaké, Côte d'Ivoire

## Abstract

The specific objectives were to identify the epidemiology of cutaneous sarcoidosis and describe the clinical and laboratory aspects of the disease. *Materials and Methods*. We performed a descriptive cross-sectional study involving 24 referred cases of cutaneous sarcoidosis in 25 years (1990–2014) collected at Venereology Dermatology Department of the University Hospital of Treichville (Abidjan) both in consultation and in hospitalization. *Results*. The hospital frequency was one case per year. The average age was 42 years, ranging from 9 to 64. The sex ratio was 1. The shortest time interval between the appearance of the skin lesion and consultation of Dermatology Department at CHU Treichville was 3 months. The elementary lesions were represented primarily by a papule (18 cases), placard (3 cases), and nodule (2 cases) and mainly sat on the face and neck in 8 cases (38%). Extra cutaneous lesions were dominated by ganglion and respiratory involvement with 5 cases each followed by musculoskeletal damage in 3 cases. Chest radiography showed abnormality in 13 cases (54%). The pulmonary function test performed in 13 patients found 7 cases (54%) having restrictive ventilatory syndrome and 6 cases (46%) being normal. A tuberculin anergy was found in 11 cases (61%).

## 1. Introduction

Sarcoidosis or besnier Boeck Schaumann (BBS) disease is a chronic granulomatous skin manifestation and a multivisceral involvement [1]. Several cases have been described by Western countries in all its aspects [2–4]. In Africa a publication was made in Western Africa, South Africa, and Tunisia [5–8]. In Ivory Coast the last study of sarcoidosis dates back to 1997 [9]. However, several gray areas persist on some aspects of sarcoidosis in black Africa. We therefore decided to conduct a study of sarcoidosis whose general objective was to help improve the treatment of cutaneous sarcoidosis among the black African. The specific objectives were to identify the epidemiology of cutaneous sarcoidosis and describe the clinical and laboratory aspects of the disease.

## 2. Materials and Methods

Our study took place at the Dermatology Department of the University Hospital of Treichville (Abidjan) which is with the Dermatology Department of the University Hospital of Bouaké, one of two reference centers for management of dermatological diseases in Ivory Coast. This was a retrospective cross-sectional study. The period from January 1, 1990, to December 31, 2014, or 25 years was concerned. All patients of all sexes and ages observed in consultation or hospitalization meeting the clinical descriptions and having a result of the histopathological examination were included. Patients with incomplete clinical record and Caucasians were not included. The collection of information was carried out with an investigation fact sheet and the following information was collected: the epidemiological characteristics and clinical and laboratory profiles. Data were analyzed with the EPI-DATA computer software.

## 3. Results

### 3.1. Epidemiological Characteristics

In the period of 25 years, 24 patients suffering from sarcoidosis were treated in the Dermatology Department. The average age was 42 years, ranging from 9 to 64. The age group 30 to 60 years was the most affected with 75% of cases. The sex ratio was 1. All socioprofessional categories were affected.

### 3.2. Clinical and Laboratory Characteristics

**Figure 1:**
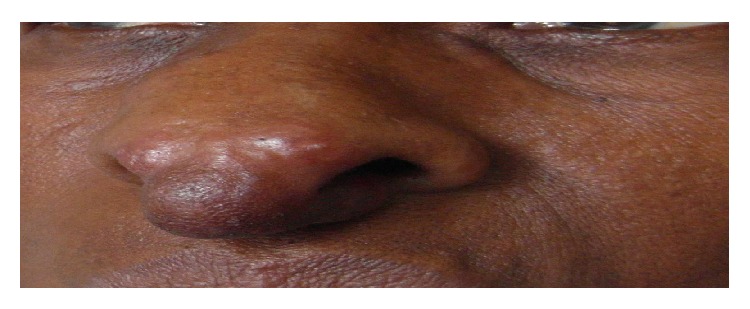
Nodule on the nose (Picture of Dr. Kouassi yao Isidore, CHU Treichville).

**Figure 2:**
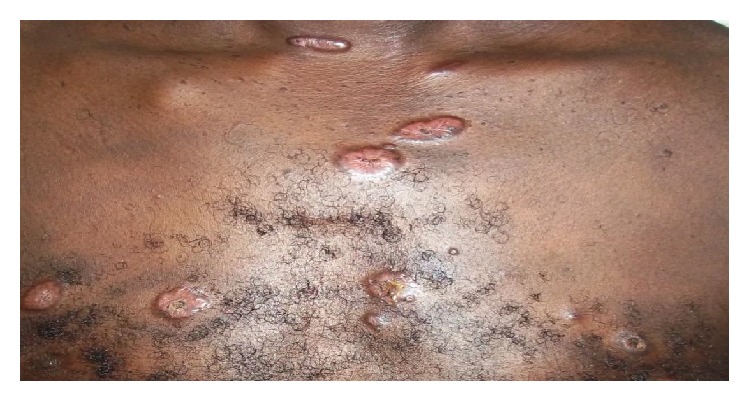
Papule of the chest (Picture of Dr. Kouassi yao Isidore, CHU Treichville).

The shortest time interval between the appearance of the skin lesion and consultation of Dermatology Department at CHU Treichville was 3 months. The mean time to consultation was 12 months. The elementary lesions were represented essentially by a papule (18 cases), a placard (3 cases), and a nodule (2 cases with one case of erythema nodosum) (Figure 1). These elementary lesions mainly sat on the face and neck in 8 cases (38%) followed by the chest in 5 cases (24%) (Figure 2) and all the body in 4 cases (19%). Lesions out of the skin were lymph nodes in 5 patients and the lungs in 5 patients too. The bones and joints were affected in 3 cases; then it was found 1 case in the eye, 1 neurological case, 1 case of spleen, and 1 case of liver. One patient had a generalized sarcoidosis with intramedullary localization leading to a bilateral flaccid paraplegia. Chest radiography showed abnormality in 13 cases (54%). The found images are shown in Table 1. The pulmonary function test performed in 13 patients found 7 cases (54%) having restrictive ventilatory syndrome and 6 cases (46%) being normal. Hematological disturbances were marked by an accelerated sedimentation rate in 4 patients and anemia in 2 cases. The tuberculin skin test was performed in 18 patients. A tuberculin anergy was found in 11 cases (61%). Serum calcium levels were normal in all patients in whom the examination was requested (9 cases), while a hypercalcium excretion was found in only 1 patient out of 2 examinations performed.

## 4. Discussion

During the study period of 25 years we have identified 24 cases. This hospital prevalence of sarcoidosis in our study is very low since 118 cases in 8 years in Spain [4], 113 cases in 13 years in Guadeloupe [3], and 118 cases in 32 years Tunisia [8] were reported. Several studies have shown that it is common in the black subject [2, 10, 11]. This low prevalence in our study could be explained by two facts. Firstly, poverty and lack of health insurance by patients limit their access to health facilities. Secondly all cases of sarcoidosis are not addressed in Dermatology Department of Treichville University Hospital because of the existence of Dermatology Department at the University Hospital of Bouaké. Young adults are the most affected with a rarity of extreme ages in our series. Our results are consistent with several African, European, American, and Asian authors [4, 7, 10, 12]. But African patients would be affected at a younger age (40 years) than patients in the Scandinavian and Asian countries with an average age of 50 years [13]. This difference could be related to life expectancy which is higher in these countries than in Africa. Sarcoidosis affects women more often than men [14]. In our series we found as many men as women with sarcoidosis. The loss and the lack of data could explain this equal frequency. Regarding history, a case of sarcoidosis was found on scarification in our study. it was a woman of thirty and she had originated in north of the Ivory Coast. Jacyk [7] also describe a similar case with a lower frequency such as a case of 54 patients with cutaneous sarcoidosis. Indeed, Olumide et al., Nigeria [6], found 43 cases of cutaneous sarcoidosis in 30 locations. These latter are all grown on cutaneous scars. These latter are all grown on cutaneous scars. The beginning of the treatment of cutaneous sarcoidosis is late. Fifty-four percent of patients said that the disease was evolving for over a year. We agree with Mahajan et al. [12] who had also found that the majority of patients present after more than one year of disease progression. Indeed the diagnosis of sarcoidosis can be difficult in forms present in early stages. Patients are then treated erroneously for other conditions such as lichen, chronic eczema, or even acne. Most often patients do not give any importance to initial lesions because there are no functional signs. The first consultation is often motivated by disfigurement. This impairs the response to therapy and therefore prognosis of the disease. No cases of sarcoidosis were discovered incidentally in our series. The diagnosis was oriented across various lesions. Among these, papules (78%) are observed and more preferably sit on the face or neck (38%). We noticed that overall general signs were almost nonexistent. In order of frequency, our results can be stacked with literature data [5, 8]. The most affected viscera during sarcoidosis in our study are the nodal unit (41.6%) and respiratory unit (33%). Niang et al. [5] and Khaled et al. [8] have made the same observation but respiratory involvement was the predominant lymph node involvement in their study. The detection of visceral involvement may be fortuitous in the paraclinical explorations. In our series, few paraclinical examinations were performed. According to data from the literature, visceral manifestations remain long asymptomatic and are discovered during diagnostic tests [15]. Erythema nodosum was found in only one patient (5%). The incidence of erythema nodosum varies across different countries and race. He found a predominance of erythema nodosum in the white matter with a frequency of 31% [16], while it is 4% among blacks [13]. Chest radiography is very important in screening for mediastinal pulmonary sarcoidosis. Some lymph nodes and miliary endothoracic origin of sarcoidosis are labeled incorrectly as a result of TB endemicity of this disease in Ivory Coast [17]. Chest radiographs were abnormal in 54% in our study and the stage I was the most common at 46%. Khaled et al. [8] found 30.1% radiographic abnormalities with a predominance of stage I or II. This high rate of pulmonary involvement could be explained in part by the poor health coverage and also by the tendency of our people to consult a doctor much later. Pathological examination was systematic in our patients. This attitude is consistent with the literature which states that the diagnosis of sarcoidosis is essentially histological [14, 18]. Laboratory tests were rarely made, which does not allow us to arrive at a judgment. Cases of anemia have no specificity. The studied immunology from the skin test revealed 61% of tuberculin anergy. Our numbers are superimposed on those in the literature [8, 19].

## 5. Conclusion

Cutaneous sarcoidosis is a relatively rare condition in Ivory Coast. It reached the youngest of all adult sex. The papules on the face and neck are the main physical signs. The lung and lymph nodes are the most commonly affected.

## Figures and Tables

**Table 1:** Radiographic images of sarcoidosis.

Images	Number	Percentage (%)
Mediastinal lymphadenopathy isolated	6	46
Reaching isolated parenchymal	5	39
Mediastinal lymphadenopathy + parenchymal damage	2	15
Total	13	100
